# The Caries Phenomenon: A Timeline from Witchcraft and Superstition to Opinions of the 1500s to Today's Science

**DOI:** 10.1155/2010/432767

**Published:** 2010-07-12

**Authors:** John D. Ruby, Charles F. Cox, Naotake Akimoto, Nobuko Meada, Yasuko Momoi

**Affiliations:** ^1^Department of Pediatric Dentistry, The University of Alabama at Birmingham, 1919 7th Ave South, Birmingham, AL 35294, USA; ^2^Department of Operative Dentistry, School of Dental Medicine, Tsurumi University, 2-1-3, Tsurumi, Tsurumi-ku, Yokohama 230-8501, Japan; ^3^Department of Microbiology, School of Dental Medicine, Tsurumi University, 2-1-3, Tsurumi, Tsurumi-ku, Yokohama 230-8501, Japan

## Abstract

This historical treatise follows the documented timeline of tooth decay into today's understanding, treatment, and teaching of caries biology. Caries has been attributed to many different causes for several millennia, however, only since the late 1900s has research revealed its complex multifactorial nature. European writers of the 1600s to 1700s held views that general health, mechanical injuries, trauma, and sudden temperature changes all caused caries—holding a common belief that decay was due to chemical agents, faulty saliva, and food particles. Until the early 1800s most writers believed that caries was due to inflammation from surrounding diseased alveolar bone. Today's science has demonstrated that caries is caused by indigenous oral microorganisms becoming a dynamic biofilm, that in the presence of fermentable sugars produce organic acids capable of dissolving inorganic enamel and dentin followed by the proteolytic destruction of collagen leaving soft infected dentin. As bacteria enter the pulp, infection follows.

## 1. The Human Tooth

The human tooth is a unique tissue composite of soft and mineralized tissues. Enamel is the hardest nonvital mineralized tissue, dentin is the hardest vital tissue and the pulp is a specialized connective tissue lined by dedicated end-stage odontoblasts that produce dentin throughout the life of the tooth, in which the pulp chamber becomes smaller over time. Each tooth is composed of unique regional diversity of anatomy, chemistry, sensory physiology, and mineral and organic components that constantly change throughout life. The interested reader is referred to Ten Cate's text for a comprehensive review of oral facial development, maturation, and growth [1].

Caries is a common human disease that only attacks vital teeth in an environment under certain oral conditions—conversely—caries does not infect a tooth once the host is dead. Studies by 19th century clinicians such as Drs. Abbot, Black, Leon Williams, Webb, Miller, and Dexter suggested a bacterial etiology to dental caries [2–11]. This paper considers the caries literature and analyzes its timeline (Table 1); erudite articles by Mandel, Newbrun, Nikiforuk, Tanzer and Zero have discussed human caries from antiquity to today [12–16]. Twentieth century scientists have clarified the intriguing complexity of the caries mosaic as an infectious disease [17–19]. The dental community realizes that the failure of the patient to remove or disrupt dental plaque biofilms or minimize frequent consumption of dietary sugars permits cariogenic bacteria to establish a dominant parasitic community.

## 2. The Antiquity of Tooth Decay

Skeletal remains are an excellent historical kymograph of human conditions. Lufkin reported that a 500,000 year-old Pleistocene skull from a human ancestor (*Pithecanthropus erectus*) from Java had severely worn teeth, however no decay was evident. He also showed a Neanderthal skull from the Paleothic era (40,000 to 25,000 year ago) with major alveolar bone loss, missing teeth, and various levels of decay in the remaining teeth; decay was recognized as a widespread disease, revealing that periodontal disease existed in almost every prehistoric race—more prevalent than decay [20].

Guerini wrote that during the reign of Hammurabi (circa 2100 B.C.) a “Code of Laws”, was left on clay tablets with judicial dictates defining fees and demanding skillful medical treatment of patients against unscrupulous mystics [21]. Before then, Ruffer discussed that most disease was attributed to the presence of unseen demons in the body or to an insult that was caused against a particular god [22]. Cuneiform tablets from that age served as the medical reference that defined special incantations to request the Babylonian god, Ea to “get hold of the worm and pull it from the offending tooth?” [21, 23].

Breasted wrote of ancient writings that provided accounts that healing of disease was linked to magic and superstitions, but had not been challenged beyond mystical thinking, until Hippocrates (460–357 BC) proclaimed that disease was due to natural causes and should be treated by means of human reason [24]. Hippocrates suggested that medicine should be dissociated from magic and witchcraft—his doctrine of disease based on humoral pathology exerted its influence on medical thought for many centuries. Stagnation of depraved juices in teeth caused dental pain [21]. “He considered affections of the teeth to depend (in part) on natural predisposition and accumulated filth and corroding action of same” [25]. Moreover, Aristotle (384–322 BC) observed a relationship of eating sweets with dental caries and proposed the question, “Why do figs, when they are soft and sweet, produce damage to teeth” [26]. 

Joris wrote of Galen (131 AD) who considered that lack of proper nutrition caused “weak, thin and brittle teeth … excessive nutrition caused inflammation to produce soft tissues and that loose teeth were the result of excess moisture that impaired the nerves” and that caries is the result of the internal accumulation of corroding humors [14, 27].

From his research of Roman cemeteries, Bressia wrote that caries was a common observation in cultures that had learned of luxury [28]. The early Roman society had elevated the Druid priesthood as a guiding influence over the health of the general population—including treatment of diseases like toothache. Ancient folklore thought that the tooth worm caused tooth decay and continued into the 1300s as seen in the writings of de Chauliac [29].

Did a tooth worm really exist? Pliny the Elder wrote of the Greek, Agatharchidas, that “people of the Red Sea suffered many strange and unheard attacks … worms and little snakes came out upon them, gnawed away their legs and arms and when touched, retracted … giving rise to unsupportable pains”. He also described the death of Pherecydes of Syros who “died of a great quantitie of creepers that came crawling out of his bodie” [30]. In 1674, Velschius described the winding of a worm on a small stick to gently remove it from the person's body [31]. In 1870, Fedechenko published the first scientific report of a 12-cm Guinea worm nematode, which he removed from a person's body, naming it *Drancunculus medinensis *[31]. The Caduceus serpent staff of Asclepius was adopted by the American Medical Association as their symbol in 1912, and could in fact represent the removal of a guinea worm with a stick by the ancients [31].

Ancient folklore described a tooth worm in holes of decay and tissues around the teeth, which caused toothache—many worldwide cultures left oral and written accounts of a tooth worm. Veracity—the truthfulness or agreement with reported facts—allows us to judge early writings. It is recorded that van Leeuwenhoek, the father of microscopy, had received three worms in a just extracted tooth—two were dead and one was alive—noting the worms were the same as ones frequenting cheese shops. When he compared live cheese-shop worms to his three, he could “not descry the least difference either in the Head or the whole Body … many old rotten cheeses had a great many little Worms in it … that upon chewing, the cheese worms insinuates themselves into the substance of the Teeth that gnawed the sensible parts, and so occasioned great pain”. Van Leeuwenhoek reported that his “wife ate heartly of old Cheese, which was seized with rottenness, and had a great many little worms in it” [32]. One of the common treatments for the tooth worm at that era was to place a few drops of *Oil of Vitriol* (sulphuric acid) into the cavity [33]. It is not surprising the ancient tooth worm theory as reported by Guy de Chauliac (1300–1368) continued into so many cultures [12].

Perhaps the Guinea worm, *Druncunculus medinensis,* that came from infected drinking water is the tooth worm. In *Dracunculiasis*, the gravid female can expel over 500,000 juvenile worms in the presence of cool water, which facilitates the release process [31]. Could it be that exposed vital pulps, which are periodically exposed to cool drinking water attract gravid females with their release of thousands of Guinea worms? This could have occurred in the ancient world where drinking water was often obtained from deep cool wells—the natural reservoir for the intermediate host of *Druncunculus medinensis*, a cyclopoid crustacean [31].

## 3. The Internal Theory of Caries: Inflammation from the Tooth Pulp

The Frenchman Paré (1510 to 1590) is credited to have almost singlehandedly elevated the respect of the dentist to a position of valued recognition in the public eyes. Paré moved away from the tooth worm theory, declaring that a toothache was due to internal forces of hot or cold humors that resulted in caries, he stated that “teeth organs alter the manner of bones, suffer inflammation and quickly suppurate to become rotten”—hence the concept of inflammation from within the tooth [34]. Kirk wrote that Pierre Fauchard (1678–1761) discredited the tooth worm theory, and was one of the first to prefer the more technical term of caries, which he thought was caused by a tumor of osseous fibers that displaced parts of the teeth causing its destruction [35].

Lufkin discussed the writings of Bondett and Jourdain who preferred the term of dental gangrene to caries [20]. Lufkin wrote that the common thought of many in the 1700s was that tooth decay was caused by death of bone and soft tissues from around or within the teeth [20]. Hunter of London expressed dissatisfaction with the term caries and preferred the term mortification, and held to the concept of the inflammation theory from internal decay, but he did not offer an alternative opinion of any substance [36].

In 1806, Fox was among the first of his contemporaries to use the term dental caries [37]. The common thought was that caries was the result of inflammation of the lining membrane (*membrane eboris*) along the pulp-dentin wall, which penetrated from the inner pulp outwards. The collective theory of many writers of that time was that nutritive factors from surrounding tissues and the pulp and were simply withheld—the pulp died and decomposed and caries proceeded through the dentin to the outer enamel surface.

In 1831, Bell of England adhered to the concept of inner inflammation, but he felt caries had a hereditary factor; he preferred the term dental gangrene to decay or caries; thinking that gangrene was a consequence of thermal changes (cold to hot), which immediately penetrated to the enamel-dentin junction, resulting in decay. Bell wrote that when dental gangrene first occurred in the bone surrounding the tooth, necrosis resulted in gangrene of the pulp resulting in its destruction and then penetrated through the dentin, and eventually to the enamel [38].

By 1825 Köecker emigrated from Germany to America and became a prominent practicing clinician in New York, he then moved to England in 1832 where he assembled his clinical observations and published his own theory of decay [39]. Köecker held similar opinions to Hunter and Fox who felt that decay was due to changes in the tooth temperature that caused inflammation. However, Köecker differed sharply with them noting from his clinical observations that decay first began on the outer enamel surface and then penetrated to the enamel-dentin junction and invaded the tubules to eventually infect the pulp tissues [39].

## 4. The External Chemical Theory of Caries Replaces the Internal Inflammation Theory

 In the late 1700s into the early 1800s, a number of colleagues from different counties—using histological preparation and stain technologies—made parallel observations that caries was caused by external chemical agents. Professor Harris of Baltimore Maryland [40], Robertson of England [41], Hope of Edinburgh [42], and Drs. Wescott and Dalyrymple [43] had collectively studied histological preparations of extracted human teeth and noted that caries could not have been caused by the mechanism of internal inflammation or from physiological changes inside the tooth. Their collective observations reported that decay was caused from outside the tooth. Robertson opined in 1835 that caries was caused by chemical disintegration of the tooth denouncing the theory of inflammation from inside the tooth. He postulated that gastric acids acted upon particles of food lodged in pits and fissures and began their destruction.

A parallel publication by Rognard of Paris in 1838 noted that caries began on the tooth surface where its effects were first seen. Rognard's clinical observations demonstrated that when extracted noncarious teeth were fixed in place of missing human teeth, caries occurred in the pits and fissures of the fixed tooth—within a few weeks [44]. Abbott described enamel caries in its earliest stage as a chemical process that dissolved the minerals that caused the breaking apart of crystals, followed by the organization of a protoplasmic mass that invaded the dentin. Abbot wrote that caries consisted of chemical demineralization and the dissolution of dentin into a “glue-giving basis-substance” around and between the tubules that breaks apart into medullary elements associated with secondary formations of micrococci and leptothrix [2–4].

Dèsirabode, the Surgeon Dentist to the King, differed with the period's collective writings on inflammation. He designated seven varieties of decay that were based on age, color, texture, damage, and other effects [45]. During those years, a great deal of confusion surrounded the idea that caries was the cause of mingling of gastric acids with mouth fluids; consequently, many simply preferred to adhere to “the chemical theory”.

Dr. Black was one of the first academics to assemble the complete pieces of the puzzle regarding the cause of caries. Several factors played to Black's favor; he had access to the current literature, plus his personal research and clinical observations gave him a unique perspective on the available written data of that day. Black wrote that tooth caries could occur when mouth fluids were habitually acidic or alkaline, and that initiation of caries was directly dependent upon lodging of food particles and gelatinous debris (plaque) at irregular pits and fissures of the tooth, followed by the fermentation of the debris with the production of acids that began the demineralization process [5]. It should be noted that for centuries, vintners had used fermentation technology to make wine, but the science of fermentation was unknown regarding the cause of dental caries. It seems Harris, Robertson, Rognard, and others had simply failed to grasp the full meaning of the relationship of caries to fermentation.

## 5. Answers Arrive from an Unlikely Source: Agricultural Chemistry

In 1840, the theory of fermentation had been fully explained by Von Liebig—an unlikely nondental scientist whose chemistry research was first presented as an oral report to the British Association for the Advancement of Science, with their full acceptance [46]. The mechanics of fermentation had been used for centuries, but it required the genius of Professor Von Liebig to present it to the scientific world in a meaningful form. Until Von Liebig, there was no understanding of fermentation in terms of chemical processes. In that era, an acceptable theory of dental caries required something more than the simple hypothesis of chemical dissolution of enamel by an acid. The acid theory was close to the true cause of caries, but the level of science of the preceding decades simply failed to understand the missing equation—bacteria. In retrospect, due to the absence of available fermentation science before Von Liebig, it is easy to understand that until the work of Louis Pasteur from 1857 to 1876 demonstrating the necessity of microbes in fermentation [47], just why the scientific understanding of bacterial fermentation causing caries was never completely understood.

When we project a few decades ahead in our scientific understanding of bacterial fermentation, we can see that Miller presented the chemo-parasitic nature of bacteria within the oral cavity and their importance in the initial cause of acid demineralization of enamel and invasion through the enamel-dentin junction to infect the tubule complex leading to destruction of collagen and other proteins [10]. It seems the actual person who might be credited with actually “FIRST” describing the exact science of caries may be left to other writers. It simply appears that its “discovery” was a collective effort by several individuals.

## 6. Defensive Capacity of Dentin against Caries

The dentist microscopist Tomes had written in 1848 from his clinical observation “the beginnings of caries, the dentine at the point of incipient disintegration becomes hypersensitive … and not just a few patients complain when parts are disturbed by the contact of foreign bodies—the dentinal tubule complex contained a life force by which the dentin was able to build a barrier against the process of disintegration and that dentine is possessed of vitality … and that vitality must have been lost before caries began and once the dentin vitality was lost in a specific area or localized point, gelatin was left to undergo gradual decomposition favored by the heat and moisture of the mouth” [48]. When Tomes applied litmus paper to the cavity of a carious tooth, it always gave a strong acid reaction that demonstrated the destruction of the mineral portions of enamel and dentin.

Professor Black wrote [49] that the 1878 studies of Leber and Rottenstein discussed that decay was a consequence of bacteria and their capacity to promote fermentation. Black showed that by treating decayed human dentin with iodine solutions, the underlying tubules showed a violet color, indicative of bacterial glycogen; he concluded that the tubules were filled with bacteria [49]. In their haste to report their observations, Leber and Rottenstein indicated that the fungus *Leptothrix buccalis* was constant in the production of caries [50]. Their observations were important to Miller as he understood the difficulties others had to contend with, but were of little use to understand the fermentation of bacteria and the cause of caries. In the late 1870s, Leber and Rottenstein showed the presence of bacteria in the tubules causing carious dentin, making a profound impact on the dental profession [50]. Milles and Underwood of London used the techniques of Koch, to verify the work of Leber and Rottenstein. A series of sterile flask experiments showed that tooth demineralization was due to acids secreted by bacteria. However, they could not accept the chemical theory of caries from acid demineralization of dentin under aseptic situations, as they placed a tooth in a closed flask with malic and butyric acid with human saliva in a meat suspension under aseptic conditions and no caries developed, finding uniform demineralization on all tooth surfaces, which did not resemble naturally occurring human caries, which was known to be more localized [51].

## 7. Science Prevails: Caries Is No Longer an Enigma

 In his small Berlin laboratory that he shared with Robert Koch, Miller observed certain bacteria could convert starch by ptyalin (amylase) to form sugar that was fermented to lactic acid [10]. Miller cited the work of Milles and Underwood who wrote that caries most likely caused decalcification as a consequence of acids secreted by oral bacteria [51]. Miller's experiments supported studies that implicated caries due to the corrosive action of lactic acid from bacteria that demineralized the mineral of enamel and dentin [10]. In hindsight, it seems that Miller's failure to recognize the true relationship of plaque bacteria to localized dental caries may have been due to his lack of clinical experience compared to that of Black [5].

Professor Black strikes an important point in his discussion that must have come to him in a “eureka” moment. He wrote in his 1884 paper *Formations of Poisons by Micro-organisms* “That fermentation is the result of the life-processes of certain forms of micro-organisms may now be accepted as a truism, and will not be argued”. He realized that fermentation was a chemical process and that a number of substances may be formed naturally by “true processes”. Having read Miller's publications and studies Black wrote “what is called fermentation by an organized fermentable agent is but the first step in true fermentation [5]”. Until that time, Miller's observations of fermentation had been mainly to study the digested agent (dentin) by lactic acid [10]. Miller had asked of the microorganisms of decay “what is its food, and in what chemical form is it delivered back after having served the purposes of the organism”. It now seems that Black was able to piece together the complex puzzle of the cause of human caries by his own and other colleague's research data.

## 8. The Final Unraveling of the Caries Phenomena

Professor Davis wrote in his textbook “the most rapid caries was of a light or white color and that the hypersensitive nature of this substrate is very high … Whereas moderately colored yellow and brown varieties are less sensitive and that the darker brown to black that represents the slow progressing form is much less sensitive when compared to normal.” Davis identified two levels of carious dentin—a superficial zone—located towards the oral surface and called infected dentin was caused by the action of lactic acid and proteases from certain bacteria that left a soft leathery substrate. The deeper zone, located towards the pulp, was called affected dentin, often referred to as secondary caries, being composed of fewer bacteria and demineralized dentin [53].

Black's use of references is an indication of his erudite nature It was obvious his depth of reading, understanding, knowledge, and forward thinking about the cause of caries for that era surpassed many others [67]. He understood that caries disintegration always begins on the enamel surface of the tooth in some pit or irregularity and that acid was formed at the very spot where caries begins. His clinical experience showed him that certain foods were associated with higher levels of caries. He grasped the importance of bacteria feeding upon lodged food particles and fermenting them to organic acids. Black had made certain personal histological observations. Caries penetration of dentin occurs by following the tubules to the pulp; his extended observations showed that pulp exposures occurred with the least destruction of dentin; “exposure of the pulp will occur … that is to say, the more perfect the development, the more complete the penetration is confined to the direction of the tubules.” He demonstrated that carious softening tended to be in isolated tubules, whereas softening of a ground section of dentin in a mineral acid was seen at its whole entirety; their appearances are distinctly different. Black also observed that in the initial carious invasion, the internal diameter of the tubules became enlarged and using an aniline dye stain, he demonstrated the tubules were occupied with bacteria. Regarding enamel caries, Black's laboratory studies demonstrated that enamel rods fell apart at the periphery and not in the rod center. His 1884 article summarized many of previous observations, “Decay of the teeth is certainly a specific disease, running a specific course, and evidently arising from a specific cause, but this cause is not yet certainly known … While there is no decay without the presence of an acid, there is not necessarily decay because of the presence of an acid [68].” It is important to realize that J. Leon Williams, a colleague of G. V. Black, also observed dental caries as an *in situ* phenomenon in teeth associated with an overlying “thick felt-like mass of acid-forming microorganisms” otherwise known as dental plaque [6–8].

## 9. A Complex Dimensional Disease: Several Layers of Carious Dentin

Using various microscopic techniques, Furrier illustrated six-zones of carious dentin: bacteria-rich, bacteria-few, pioneer-bacteria, turbid-layer, transparent and a vital reaction layer. However, from a clinical point of view, tactile discrimination of caries varied from clinician to clinician due to its softness [69]. The issue of caries discrimination was solved by Professor Fusayama and Terachima, using an *in vivo* stain. They demonstrated that softened carious dentin is composed of two layers [57]. Their research demonstrated an outer infected carious zone just below the enamel-dentin junction densely populated with facultative and anaerobic bacteria that secrete (1) organic acids capable of dissolving hydroxyapatite crystals, and (2) proteases that degrade collagen and other proteins causing detachment of apatite crystals leaving the once solid substrate to simply collapse on itself. This outer infected caries is completely dead, with no capacity to register any sensitivity to tactile or thermal stimuli and is not physiologically capable of remineralization. This fact makes its removal clinically painless as no anesthesia is necessary. The deeper affected carious dentin is generally 1,000 to 2,500 *μ*m thick and generally contains only a few pioneer bacteria. It is somewhat softened due to organic acids dissolving the mineral rich crystals without proteases damaging the organic proteins [57]. This deeper carious zone is vital with a sensory capacity to respond to various stimuli. Once the clinician reaches this vital layer with minimally invasive instrumentation, they realize when to stop instrumentation as the underlying affected tubule complex is physiologically capable of remineralization with crystals that fill the lumen of dentinal tubules to become sclerotic [59, 65]. Importantly, the application of these principles has evolved into the therapeutic use of indirect pulp capping [70–72] and stepwise excavation [73–75] for the conservative preservation of the vital dental pulp during clinical caries removal as long as a “bacteriometic” seal can be maintained [61, 64, 76, 77]. 

“An Ounce Of Prevention Is Worth A Pound of Cure” [78]. This expression from Benjamin Franklin (1706–1790) means it is better to avoid problems in the first place, rather than trying to fix them once they arise. In a 1886 lecture to students, G. V. Black stated “The day is surely coming, and perhaps within the lifetime of you young men before me, when we will be engaged in practicing preventive, rather than reparative, dentistry” [79]. We wonder what Black would think if he realized that most of today's dental schools throughout the world still teach a restorative focused curriculum; rather than a series of preventive courses? Since the 1970s, our profession has witnessed the introduction of caries detectors, acid etchants, glass ionomers and composites that seem more suited to minimal intervention than Black's extension for prevention concepts of amalgam placement.

The addition of fluoride to public water has proved effective to reduce caries in human dentitions [80]; postdevelopmental use of fluoride is known to cause a significant reduction in caries through topical interaction with surface enamel and dentin throughout life [60, 66, 81]. Other measures have shown that an alteration or reduction of dietary sugars also results in a major decrease of caries in experimental animal models [82, 83] and humans [55, 58].

It is interesting to pause and reflect on dental research since mid-1800. Once caries was known to begin on the external tooth surface and proceed inwards, the dental profession gained recognition amongst the worldwide populace. As the science of caries prevailed, the tooth worm faded into oblivion. New devices and technologies emerged in parallel fashion and became used in the laboratories of clinicians who were searching for answers to the biology of the tooth and caries.

North American notables such as Harris (1806–1860), Black (1836–1915), Webb (1844–1883), Williams (1852–1932), and Miller (1853–1907) all shared very common childhood experiences [5, 9, 10, 40]. They were not born of nobility or gentry, but grew up in humble rural surroundings and learned of life by spending long hours in the pursuit of Nature. American cultural history records that almost every home contained the popular textbook of the day of Comstock's Philosophy for family reading and group discussions after dinner time in the evening [84]. Each of these individuals had a similar introduction to dentistry and study, they used their own personal finances; no governmental agency dispensed research funds for their research. They pursued answers to questions that had evaded other colleagues and published their findings because they wanted to make sure new knowledge was available to colleagues worldwide. There was no academic pressure to publish or perish.

## 10. Remaining Challenges

Where should we go from here? It seems that much of the above information, although still available in the dental literature, remains somewhat lost in the academic teaching of caries for today's dental students. A fundamental knowledge of dental caries and the pulpal response to this bacterial insult remains illusive to many of today's clinicians and educators. Since the 1880s, we have learned that bacteria are the cause of caries [15] as a dynamic biofilm (dental plaque) [62, 63], and that bacteria are essential for pulpal disease [56]. Restorative procedures and devices have been developed to identify and remove caries. Has our current cosmetic-restorative era failed us? Are today's dental students integrating the appropriate clinical and scientific information for caries risk assessment, minimal intervention in caries removal, preservation of the vital pulp, and total prevention of dental decay within the human dentition? Thanks to the personal curiosity and initial research efforts of Harris, Webb, Black, Williams, Miller, and other colleagues of the late 1880s, our dental community now recognizes the cause of caries. The authors, again, remind the readers of Professor G. V. Black's challenge from 1886, “The day is surely coming, and perhaps within the lifetime of you young men before me, when we will be engaged in practicing preventive, rather than reparative, dentistry.” [79]. The time is Now as we travel along this timeline from the past to the future. Our scientific community has made enormous advances in molecular biology to further our understanding of dental caries as a biological phenomenon [85–87]. We must integrate our current discoveries and past knowledge base into clinical practice. Let us not only prevent dental caries at all levels, but also preserve the vital dental pulp.

## Figures and Tables

**Table 1:** The caries phenomenon timeline.

Date	Clinical/Scientist	Observations
40.000–25,000 BC		Decay and alveolar bone loss is evident in the jaws of Neanderthal skulls from the Paleolithic Era [20].
22,000 BC		Decay of teeth and bone loss on Cro-Magnon jaws from the Paleolithic Period showed most lesions were located at or along the cement-enamel junction [20].
2,100 BC		Clay tablets from Assyria asked the goddess Ea to place the tooth worm between the teeth and jaw bone to destroy the blood and strength of the teeth [21, 23].
1,500 BC		Oracle bones of the Shang Dynasty of China showed characters that mentioned a tooth worm that invaded the mouth and teeth [21].
460–377 BC	Hippocrates	Greek Father of Medicine whose doctrine of disease was based on humoral pathology: stagnation of depraved juices in teeth caused pain. He discredited disease being caused by magic or mythology [21, 24].
384-322 BC	Aristotle	Greek philosopher who observed that sweet foods such as soft figs and dates caused a sticky film on the tooth that led to putrification and tooth decay [26].
200 BC	Agatharchidas	People of the Red Sea suffered and died from small worms that gnawed away on many body tissues [30].
62 AD	Pliny the Elder	Wrote that his friend Pherercydes of Syros died from creepers that crawled from his mouth and body [30].
129–200/217 AD	Galen of Pergamum	A Greek physician who believed that poor nutrition caused weak, thin, and brittle teeth; accumulation of internal corroding humors caused caries [14, 27].
1300–1368 AD	Guy de Chauliac	Believed the tooth worm existed and was responsible for tooth decay. He suggested fumigation with leek, onion, and Henbane to cure the persons tooth pain [29].
1525 AD	Ambroise Paré	Internal life forces from within the body and teeth caused decay. He discredited the tooth worm idea [34].
1684 AD	Antonie van Leeuwenhoek	Observed many small spinning microorganisms from mouth spittle,which he called animalcules [47].
1700 AD	Bondette and Jourdain	They called caries a dental gangrene that was caused by tissue inflammation and death of the bone around the tooth neck [20].
1700 AD	Antonie van Leeuwenhoek	Wrote to the Royal London Society that he took live tooth worms from corrupt teeth of his wife, noting they were the same as living cheese-worms that were found from a cheese shop [32].
1728 AD	Pierre Fauchard	Considered to be The Father of Modern Dentistry, discredited the tooth worm theory, and thought dental caries was caused by a tumor of osseous fibers [20, 35].
1780 AD	John Hunter	Preferred the term mortification to caries, and believed the source of decay was due to an imbalance of internal forces that caused inflamation and pulp disease [36].
1798 AD	T. Charles Hope	He believed caries was due to external forces, and dismissed the internal tooth inflammation theory [42].
1806 AD	Joseph Fox	Preferred the term caries. He believed tooth inflammation was due to internal injury of the lining membrane along the pulp-dentin wall [37].
1831 AD	Thomas Bell	Believed that caries had a hereditary component [38].
1835 AD	William Robertson	Caries was due to the chemical disintegration on the outside of the tooth. He denounced internal factors [41].
1838 AD	M. Rognard	Believed that caries began in pits and fissures of the crown on the outside of the tooth [44].
1841 AD	M. A. Dèsirabode	Designated seven stages of tooth decay [45].
1841 AD	Levi Spear Parmly	The first advocate of oral hygiene for the patient [52].
1842 AD	Leonard Köecker	Believed that tooth caries was due to internal inflammation from rapid temperature changes [39].
1843 AD	A. Wescott and J. W. Dalyrymple	English clinicians who believed tooth decay was caused by external forces of the oral environment [43].
1847 AD	Justis von Liebig	Described fermentation as a chemical process [46].
1848 AD	John Tomes	Believed that incipient caries caused mineral disintegration that led to tooth hypersensitivity [48].
1855 AD	Chapin A. Harris	Early American educator who believed that caries was due to external factors of the oral environment [40].
1861 AD	Louis Pasteur	Demonstrated that fermentations are “vital processes” requiring microorganisms [47].
1878 AD	T. Leber and J. W. Rottenstein	Believed that caries was due to bacterial fermentation of food debris, and oral fluids that led to the presence of bacteria in dentin tubules [50].
1879 AD	Frank Abbott	Believed that caries was due to a chemical process that dissolved tooth minerals, followed by the formation and organization of a protoplasmic gelatinous mass [2–4].
1881 AD	G. A. Milles and A. S. Underwood	Caries was most likely due to demineralization by organic acids produced by bacteria [51].
1884 AD	Greene Vardiman Black	First to assemble the caries puzzle that involved food debris, gelatinous debris, and acids, which caused demineralization leading to the initial caries lesion [5].
1890 AD	Willoughby D. Miller	Caries was due to corrosive actions of lactic acid from bacteria that caused enamel lesions [10].
1897 AD	John Leon Williams	Decayed human teeth showed a dense felt-like mass of acid-forming microorganisms, dental plaque, that exerted its chemical influence upon calcified tissues [6–8].
1923 AD	W. Clyde Davis	Identified a soft superficial carious zone with many bacteria and deeper caries zone with fewer bacteria and some demineralization [53].
1940 AD	R. M. Stephan	*In situ* changes in dental plaque biofilm pH in the presence of sugar [54].
1954 AD	B. E. Gustafsson	Frequency of sugar consumption in institutionalized children (Vipeholm) related to caries experience [55].
1955 AD	Frank J. Orland	Demonstrated that caries did not develop in germ-free rats [15].
1960 AD	Ron Fitzgerald and Paul Keyes	They demonstrated the etiological role of specific streptococci in the caries process making it an infectious and transmissible disease [15].
1965 AD	Sam Kakehashi	Demonstrated bacteria are necessary for pulpal inflammation or necrosis using germ-free animals [56].
1972 AD	Takao Fusayama and S. Terachima	Showed clinical discrimination of two layers of carious dentin with a biological stain that provided distinct visual differentiation of infected and affected layers [57].
1975 AD	A. Scheinin and K. K. Makinen	Turku study indicated that replacement of sugar with xylitol decreased caries experience [58].
1978 AD	Maury Massler	Showed the clinical importance for the dentist to differentiate the outer infected active carious dentin from the deeper arrested carious dentin [59].
1980 AD	Theodore Koulourides	Lesion consolidation with remineralization and rehardening of enamel in calcifying solutions containing fluoride [60].
1981 AD	Martin Brännström	Bacterial microleakage into dentin and pulp causes recurrent decay, pulp inflammation and necrosis [61].
1986 AD	Walter J. Loesche	Developed the “specific plaque hypothesis” that stated caries was an acidogenic bacterial infection caused by mutans streptococci and lactobacilli species [62].
1994 AD	Philip D. Marsh	Developed the “ecological plaque hypothesis” to describe the dynamic relationship within plaque biofilm consortiums where low pH selects for the growth of cariogenic microorganisms [63].
1998 AD	Eva. J. Mertz-Fairhurst et al.	Ten-year clinical outcome study of carious lesions with sealed dentin showed arrested lesion progression with no more clinical pulp failures when compared to the control group with conventional caries removal [64].
2004 AD	Edwina A. M. Kidd	Metabolic activity in the human plaque biofilm is the all-important driving force behind any loss of mineral from the tooth or cavity surface and resultant pulp inflammation [65].
2009 AD	Eric C. Reynolds	Concluded that calcium phosphate-based remineralization technologies showed promising adjunctive treatments to fluoride therapy in early caries management [66].
